# The Effect of Autistic Traits on Social Orienting in Typically Developing Individuals

**DOI:** 10.3389/fpsyg.2020.00794

**Published:** 2020-04-23

**Authors:** Guoyao Lin, Yanling Cui, Jiajing Zeng, Liang Huang

**Affiliations:** ^1^Center for Studies of Psychological Application, School of Psychology, South China Normal University, Guangzhou, China; ^2^Fujian Key Laboratory of Applied Cognition and Personality, School of Educational Science, Minnan Normal University, Zhangzhou, China

**Keywords:** autism spectrum disorder, autistic traits, attention orienting, social cues, non-social cues

## Abstract

Autism spectrum disorder (ASD) is a complex neurodevelopmental disorder characterized by wide ranging and heterogeneous changes in social and cognitive abilities, including deficits in orienting attention during early processing of stimuli. Investigators have found that there is a continuum of autism-like traits in the general population, suggesting that these autistic traits may be examined in the absence of clinically diagnosed autism. To provide evidence for the continuum of autistic traits in terms of social attention and to provide insights into social attention deficits in people with autism, the current study was conducted to examine the effect of autistic traits of typically developing individuals on social orienting using a spatial cueing paradigm. The typically developing individuals who participated in this study were divided into high autistic traits (HA) and low autistic traits groups using the Autism Quotient scale. All participants completed a spatial cueing task in which social cues (gaze) and non-social cues (arrow) were presented under different cue predictability conditions (predictive vs. non-predictive) with different SOAs (100 ms vs. 400 ms). The results showed that compared to low autistic individuals, high autistic individuals had less benefit from non-predictive social cues but greater benefit from non-social ones, providing evidence that such spatial attention impairment in high autistic individuals is specific to the social domain. Interestingly, the smaller benefit from non-predictive social cues in high autistic individuals was shown only in the 400 ms condition, not in the 100 ms condition, suggesting that their difficulties in orienting to non-predictive social cues may be caused by a deficiency in spontaneously effortful control processing.

## Introduction

Autism spectrum disorder (ASD) is a complex neurodevelopmental disorder characterized by impaired social communication and interaction skills combined with repetitive and stereotyped patterns of interests and behaviors (American Psychiatric Association, 2013). Research supports that autism is situated at or near the extreme end of a continuum of autistic traits, especially with respect to deficits in the social and communicative behavior seen in the general population (Wing and Gould, 1979; Baron-Cohen, 1995; Baron-Cohen et al., 2001b; Constantino and Todd, 2003; Constantino, 2011). Based on this view, Baron-Cohen et al. (2001b) developed an instrument (Autism Spectrum Quotient, AQ) to quickly quantify the autistic traits of any given individual in the general population. Since then, a growing number of findings have indicated that autistic traits as measured by the AQ correlate positively with autism-like impairment of performance in typically developing adults on social tasks, such as mind reading (Baron-Cohen et al., 2001a), face recognition (Valla et al., 2013), social learning (Hudson et al., 2012; Sevgi et al., 2019), and self-referential cognition (Lombardo et al., 2007). However, previous studies have focused on the relationship between autistic traits and the relatively high-level components of social cognition, and less on the relationship between autistic traits and social attention, which is the crucial front-end to all higher-level social cognition (Puce and Bertenthal, 2015). In the present study, we investigated the relation between attentional orienting to social cues and autistic traits in neurotypical individuals, with a view to enriching our understanding of the autistic traits continuum and to provide insight into the attention-orienting deficit in the clinically-affected ASD population.

### The Important Role of Social Attention and Its Relationship With ASD

Social attention is generally defined as the selection and encoding of social cues by an observer (Happé et al., 2017). Social attention plays an extremely important role in social information processing and social capacity development (Puce and Bertenthal, 2015). For example, soon after birth human infants have an attention preference for other people’s eyes (Farroni et al., 2002). This preference is one of the social adaptation mechanisms selected by human beings in the long evolutionary process and plays a fundamental role in the process of socialization (Emery, 2000). With the psychological development of children, the manifestation of social attention can be used as a window to reflect development and change in social ability (Frank et al., 2012). Compared to those with typical development, ASD individuals pay insufficient attention to social information in the early stage of development, which results in a lack of sufficient social learning and, further, in deviation from the normal development of social cognition and social ability.

However, researchers have different views on the mechanism of social attention deficit in ASD individuals. According to the general perspective in the domain, early social communication requires individuals to quickly orient and shift attention to different stimuli. This capacity is deficient in persons with ASD, thus leading to social capacity impairment (Harris et al., 1999). In contrast, the domain-specific viewpoint holds that the attention-orientation deficit in ASD is restricted to socially relevant cues, which results in a selective attenuation of the tendency to initiate social attention orientation (Mundy, 1995). In order to resolve the dispute between these two viewpoints, researchers have carried out many experimental studies, especially on social orientation using the spatial cue paradigm.

### Research on Social Orienting Deficiency in ASD Based on the Spatial Cue Paradigm

The spatial cue paradigm is the attention research paradigm proposed by Posner (1980). In this paradigm, the difference in response time to a target between the invalid cue condition (i.e., the cued position is inconsistent with the position of the target) and the valid cue condition (i.e., the cued position is consistent with the position of the target) is called the cueing effect, which reflects the degree of attention orienting. By comparing the social (gaze) and non-social (arrow) cueing effects in this paradigm, researchers have attempted to examine whether the attention orienting deficit in the ASD population is specific to the social domain (Senju et al., 2004; Ristic et al., 2005; Vlamings et al., 2005; Goldberg et al., 2008). However, the results of these studies are quite variable. Some studies found that ASD individuals were able to orient to social cues as well as to non-social cues (Senju et al., 2004; Vlamings et al., 2005), while others found that ASD individuals oriented to social cues abnormally (Ristic et al., 2005; Goldberg et al., 2008).

The variation in the results of these prior studies may have been caused by inconsistencies in the manipulation of the experimental variables. First, the cue duration and the stimulus onset asynchrony (SOA) between the cue and the target might affect the experimental results in the spatial cue paradigm (Green and Woldorff, 2012; Green et al., 2013). In addition, cue predictability (i.e., the probability that the target appears in the direction indicated by the cue) might also affect the experimental results. For example, Ristic et al. (2005) found that when the cue predictability was 80%, ASD subjects showed the same level of social orienting as normally developing subjects, whereas when the cue predictability was 50%, they showed insufficient social orienting. Different manipulations of the experimental variables may have caused different types of social orienting. A short SOA or low cue predictability may trigger reflexive social orienting (Ristic et al., 2002; Swettenham et al., 2003), in which case ASD individuals may be inclined to perform abnormally (Morrisey et al., 2018); In contrast, a long SOA or high cue predictability may trigger controlled social orienting, in which case ASD individuals might perform normally (Morrisey et al., 2018).

### The Present Study

To sum up, although researchers have conducted a large number of studies on social orienting in the ASD population by using the spatial cuing paradigm, it is still not clear whether social orienting deficiency in ASD is domain-general or social-specific, and whether this deficiency is caused by insufficient reflexive orientation. The relationship between social orienting deficiency and autistic traits is also worth exploring to assess the validity of the hypothesis of a continuum of autistic traits.

Therefore, the present study aimed to explore the social orienting deficiency and its relationship with autistic traits in neurotypical population. Like the study of Morrisey et al. (2018), we used social cues (gaze) and non-social cues (arrow) in a spatial cuing paradigm to compare attention orienting between social and non-social domains, and manipulated cue predictability (50% vs. 80%) and the SOA (100 ms vs. 400 ms) between the cue and the target to trigger and compare reflexive and controlled orienting. But unlike Morrisey et al. (2018), we investigated these questions in neurotypical population rather in ASD population, with the aim to assess the validity of the hypothesis of a continuum of autistic traits.

It is worth mentioning that Bayliss et al. (2005) have preliminarily investigated the similar problem as our study and reported that autistic trait scores in the neural-typical population were negatively correlated with the eye-gaze cue effect but not with the arrow cue effect. But in their study, there were still several methodological problems. Firstly, the two types of cue effect were compared between experiments rather within a same experiment, so that some disturbing variables such as differences between subjects could not be excluded. Secondly, the gaze cues used in their study were cartoon faces rather than real faces, which might reduce the ecological validity of materials (Chevallier et al., 2015). Thirdly, the long cue duration (i.e., the cue were presented until respond) in their study might brought interference effect similar to Simon effect (Green and Woldorff, 2012; Green et al., 2013). We tried to avoid these methodological problems in the present study by comparing the two types of social orienting in one experiment, using real face as social cue, and presenting the cues under short cue duration (i.e., the cue disappeared before the target was presented).

Generally speaking, in the present study we investigated the relationship between attentional orienting to social cues and autistic traits in neurotypical individuals, with a view to providing insight into social orienting deficiency in the ASD population and to examine the hypothesis of a continuum of autistic traits in the social attention domain. Firstly, we aimed to explore whether the level of autistic traits influences the effect of social cues and non-social cues differently. We predicted that for those with high autistic traits (HA), if the attention orientating deficits are specific to social cues, there will be no impaired orientation to non-social arrow cues in contrast to gaze cues. Otherwise, according to the domain-general viewpoint, attention orienting deficits in those with HA will not differ between cue types. Secondly, we aimed to explore whether autistic traits have different effects on reflexive and controlled social orienting. If individuals with HA show attention orienting deficits only to non-predictive gaze cues in the short SOA condition, we could conclude that the attention orienting deficits in individuals with HA derives from a reflexive rather than a voluntary social attention orientation. In contrast, if the difference between groups is not influenced by cue predictability and the SOA manipulation, we could not support the reflexive nature of the attention orienting deficits in individuals with HA. According to the previous studies (Bayliss et al., 2005; Morrisey et al., 2018), we hypothesized that autistic traits of neurotypical individuals would only selectively influenced social orienting but not non-social orienting, and this effect was resulted from voluntary orienting rather than reflexive orienting.

## Method

### Participants

Forty-seven college students (26 male and 21 female) from South China Normal University participated in the experiment. They were recruited through various social platforms, such as campus BBS, and were paid for their participating. The mean age of the participants was 20.77 years (*SD* = 1.07). All participants were right-handed and reported normal vision or corrected-to-normal vision. The participants provided informed consent to participate in the research study, which was approved by the local institutional review board.

Before the experimental task, each participant completed the Autism Spectrum Quotient (AQ: Baron-Cohen et al., 2001b), which was developed to assess the level of autistic traits in the general population. The AQ produces scores in five domains: social skills, attention switching, attention to detail, communication, and imagination. It includes 10 items for each domain and each item is scored 0 or 1. The total score on the AQ ranges from 0 to 50, with higher scores connoting a higher level of autistic traits. The AQ was normalized for use in the Chinese population, and the traditional Chinese version of the AQ was shown to have good coefficients for internal consistency and test-retest reliability (Liu, 2008). The simplified Chinese version of the AQ used in this study was also shown to be a reliable instrument for quantifying autistic traits in both clinical and non-clinical samples in mainland China (Zhang et al., 2016). The AQ scores of participants in this study ranged from 13 to 33 (*M* = 23.09, *SD* = 5.24). Using a median-split, all participants were divided into a low-AQ group (LA, *n* = 24, *M* = 18.45, *SD* = 3.11) and a high-AQ group (HA, *n* = 23, *M* = 27.16, *SD* = 2.69).

### Procedure

The experimental procedure was programmed using E-Prime 2.0 software (Psychology Software Tools, Pittsburgh, PA, United States), and the stimuli were presented on a personal computer with an LCD screen. Participants were tested individually in a quiet room, sitting in a chair and viewing the screen 60 cm away.

There were four blocks in the experiment: predictive social cue (eye gaze) block, non-predictive social cue (eye gaze) block, predictive non-social cue (arrow) block, and non-predictive non-social cue (arrow) block. Each participant completed all four blocks, and the order was counterbalanced across participants. In each block there were 10 practice trials and 120 experimental trials. In the predictive block, 80% (96) of the experimental trials were valid trials (targets appeared on the cued side), 10% (12) were invalid trials (targets appeared opposite to the cued side), and the other 10% (12) were catch trials (no target, no response). In the non-predictive block, the ratios of valid trials, invalid trials, and catch trials were 45% (54), 45% (54) and 10% (12), respectively. Participants were informed of the ratio of valid trials before each block.

**FIGURE 1:**
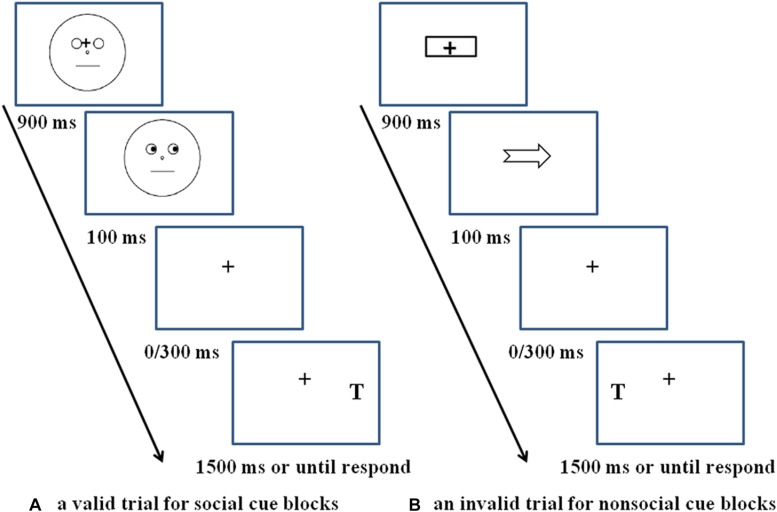
Schematic of an experimental trial. **(A)** A valid trial for social cue blocks, **(B)** an invalid trial for non-social cue blocks. Schematic faces in panel **(A)** are only used for depiction purposes. The actual stimuli used in the experiment was a real face picture from Chinese facial affective picture system (see section Procedure) with permission from the copyright holders of the database.

For each experimental trial (see Figure 1A) with social cue blocks, the procedure began with a realistic face picture (4.5° × 8.6°), the original version of which is obtained from the Chinese facial affective picture system (Gong et al., 2011), presented at the center of the computer screen. The face picture had a black central fixation cross (1.5° × 1.5°) between the eyes, and the iris and pupils were masked by the same color as the whites of the eyes to avoid the emergence of a dynamic effect, which means the eyes remained static throughout the whole trial. After 900 ms later, an eye gaze cue (the original unmasked version of the just presented face picture) was presented with the eyes looking left or right, indicating the position of the target. The eye gaze cue was presented for 100 ms and then disappeared from the screen, leaving only the fixation point for 0 ms (100 ms SOA condition) or 300 ms (400 ms SOA condition). Then a target letter T (1.5° × 2.5°) might present on the left or right side of the fixation point, regardless of whether the trial was valid or invalid; participants needed to press the space bar with the left or right index finger, respectively. If T was not presented (catch trials), no response was needed. The next trial began immediately after the response, or 1,500 ms later if there was no response.

For the non-social cue blocks (see Figure 1B), the procedure was exactly the same as for the social cue blocks, except that the social gaze cues were replaced by the non-social arrow cues (4.5°× 2.5°).

## Results

### Descriptive Statistical Analyses

**FIGURE 2:**
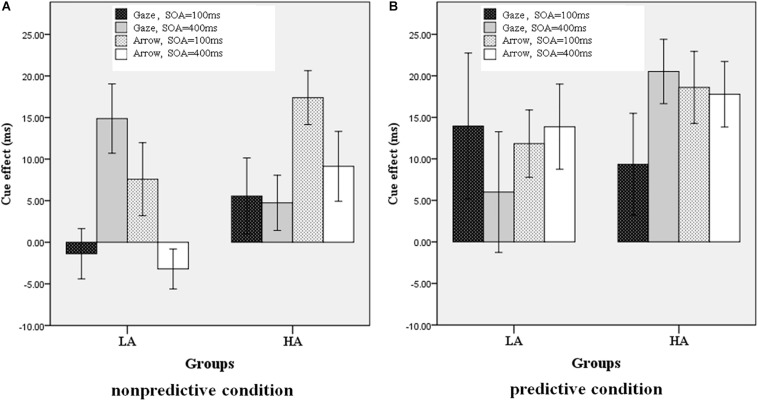
Cue effect in all conditions for the LA and HA groups. **(A)** Non-predictive condition, **(B)** predictive condition.

We used SPSS 18.0 (PASW Statistic 18.0) for the statistical analyses. Trials with response errors or with extreme reaction time (RT) values (less than 100 ms or greater than 3 *SD* above the average RT in each condition) were excluded from the formal analyses (2.46% of trials). Because the accuracy indexes were all near 100% in all conditions, they were also excluded from the formal analyses to avoid ceiling effects. The mean RTs under all conditions for the LA and HA groups are shown in Table 1. The cue effect was derived by subtracting the RT in the valid condition from the RT in the invalid condition, as shown in Figure 2.

**TABLE 1:** Mean reaction times (*SD*) in all conditions for the LA and HA groups.

Condition			LA group (*n* = 24)		HA group (*n* = 23)	
Cue type	Cue predictability (%)	SOA (ms)	Valid trials	Invalid trials	Valid trials	Invalid trials
Gaze	50	100	358 (36)	357 (38)	367 (52)	373 (54)
		400	312 (39)	327 (37)	319 (51)	324 (47)
	80	100	361 (36)	375 (61)	369 (62)	378 (78)
		400	317 (38)	323 (35)	323 (67)	343 (71)
Arrow	50	100	347 (39)	355 (39)	342 (41)	360 (40)
		400	317 (32)	314 (30)	309 (41)	318 (40)
	80	100	345 (39)	356 (42)	346 (33)	364 (40)
		400	309 (30)	323 (33)	305 (34)	323 (46)

### Inferential Statistical Analyses

In order to test the influence of the four independent variables on the cue effect, a four-factor repeated measures ANOVA was conducted on the cue effect, with cue type (gaze, arrow), cue predictability (predictive, non-predictive), and SOA (100, 400 ms) as the within-subjects variables, and group (LA, HA) as the between-subjects variable. There was a significant interaction among the four independent variables, *F*(1, 45) = 5.722, *p* = 0.021, η^2^= 0.113. The main effect of cue predictability was also significant, [*F*(1, 45) = 3.13, *p* = 0.10], with a larger cue effect for high cue predictability (80%) than for low cue predictability (50%). The other main effects and interactions were not significant (*p* > 0.05).

**FIGURE 3:**
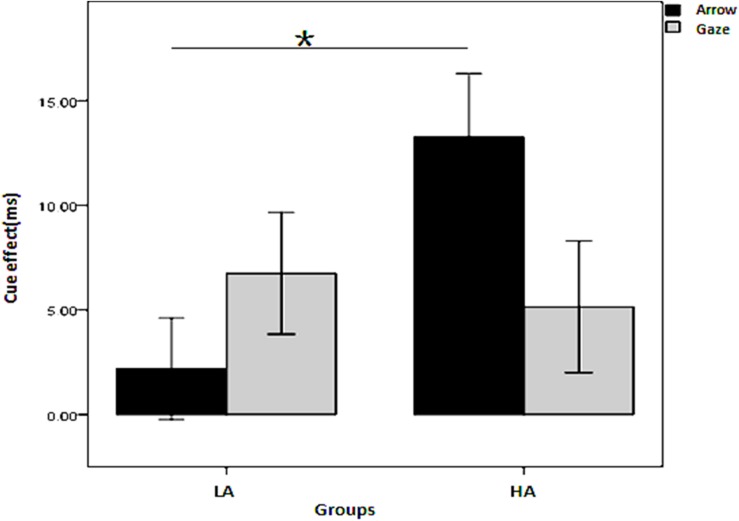
Interaction between group and cue type on the cue effect in the non-predictive condition. *Indicates significant difference between the two conditions.

To simplify and examine the interaction of the effect of the four independent variables on the cue effect, a three-factor repeated measures ANOVA was conducted on the cue effect with cue type and SOA as the within-subjects variables and group (LA, HA) as the between-subjects variable for the two cue predictability conditions, respectively. When the cue predictability was 80%, all the main effects and interactions were non-significant (*p* > 0.05). When the cue predictability was 50%, there was a significant interaction among the three independent variables, *F*(1, 45) = 4.386, *p* = 0.042, η^2^= 0.089; the interaction between groups and cue types was also significant, [*F*(1, 45) = 13.563, *p* = 0.001, η^2^= 0.232]. As shown in Figure 3, the simple effects analysis revealed that the cue effect for the HA group was larger than for the LA group in the arrow condition (*p* = 0.006) but not in the gaze condition (*p* = 0.711).

**FIGURE 4:**
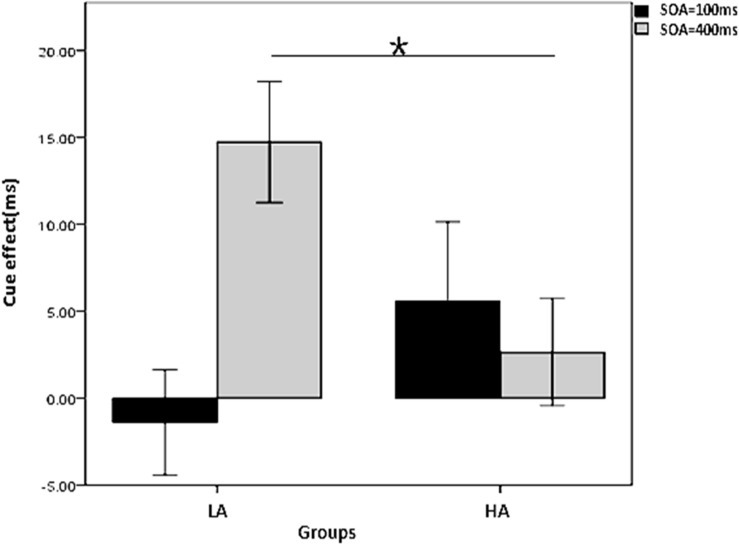
Interaction between group and SOA on the gaze cue effect in the non-predictive condition. *Indicates significant difference between the two conditions.

To further explore the interaction of group, cue type, and SOA on the cue effect with 50% cue predictability, a two-factor repeated measures ANOVA was conducted on the cue effect, with SOA as the within-subjects variable and group (LA, HA) as the between-subjects variable for the two cue types, respectively. The results in Figure 4 show that (1) in the arrow cue condition, there is a significant main effect for group, with a larger cue effect for the HA group than the LA group (*p =* 0.006); (2) in the gaze cue condition, there is a significant interaction between group and SOA (*p* = 0.024); the simple effects analysis shows that the cue effect is larger for the LA group than for the HA group; for the HA group it is significant in the 400 ms SOA condition (*p* = 0.013) but not in the 100 ms SOA condition (*p* = 0.209).

## Discussion

This is the first detailed examination of attention orienting to social and non-social cues in typically developing adults who are low or high in autistic traits. The study provides evidence supporting domain-specific social attention deficit in individuals with HA. Specifically, the HA group showed deficiency of attention orienting only for social cue (gaze) processing, and even an advantage for non-social cue (arrow) processing. Further, we found that the deficiency of social orienting in the HA group manifested only in the processing of non-predictive social cues (i.e., 50% cue predictability), not in the processing of predictive social cues (i.e., 80% cue predictability), indicating that this deficiency was related to lack of spontaneity. Furthermore, this deficiency was found in the late processing (i.e., under 400 ms SOA) of non-predictive social cues, but not in the early processing (i.e., under 100 ms SOA) of such cues, suggesting that this deficiency was related to effortful control processing after spontaneous initiation.

### Different Effects of Autistic Traits on Social Orienting and Non-social Orienting

The first concern of this study was whether individuals in the typical developing population with HA show deficiency of attention orienting compared with those with mild autistic traits; and if so, is this deficiency domain general or social domain specific? The results show that the HA group showed impaired orienting only to non-predictive gaze cues, whereas they performed even better than the LA subjects with the non-predictive arrow cues. These results suggest that for individuals with HA, the attention orienting defect was present only in the processing of social cues, but not in the processing of non-social cues, which supports the social domain specific perspective. Similar to our study, Bayliss et al. (2005) reported that autistic trait scores in the neural-typical population were negatively correlated with the eye-gaze cue effect. However, Bayliss et al. (2005) found no correlation between autistic traits and the arrow cue effect, whereas we found in our study that subjects with HA had an advantage in processing arrow cues, providing stronger evidence for the social domain specific perspective.

It was an interesting finding that individuals with high autism traits showed an advantage in attention orientation to non-social cues. Based on the extreme male-brain theory of autism, Bayliss et al. (2005) also expected autistic traits to be positively correlated with the arrow cue effect; but they did not find what they had expected, perhaps because the sample size (18 males and 18 females) in the study was relatively small. However, in our study we found that subjects with high autism traits showed an enhanced arrow cueing effect, which is consistent with the hypothesis from the extreme male-brain theory; that is, individuals with HA have the advantage of systemizing (i.e., the capacity to predict and respond to an abstract symbol-like arrow). This advantage may help individuals with HA overcome a lack of social orienting and thus have normal social functioning (Baron-Cohen et al., 2005). A similar compensation effect has also been found in ASD individuals, who can compensate for the deficiency of automated processing by effortful control in facial expression processing (Harms et al., 2010), which is closely related to the second concern of this study.

### Different Effects of Autistic Traits on Reflexive and Controlled Social Orienting

The second concern of this study was whether autistic traits have different effects on reflexive and controlled social attention orientation. The results show that although the HA group demonstrated insufficiency in orienting to a non-predictive gaze compared to the LA group, they were able to orient to a predictive gaze as successfully as the mildly autistic subjects. These results suggest that autistic traits affect only reflexive social orienting, not controlled social orienting. Consistent with our study, Ristic et al. (2005) found that children with autism showed insufficient orienting to a non-predictive gaze, but they could orient to a highly predictive gaze as well as children with typical development. We can therefore infer that the negative effect of autistic traits on reflexive social orienting is consistent across clinical diagnoses. However, autistic traits selectively affect only reflexive social orienting, not controlled social orienting. So individuals with HA may compensate for the lack of reflexive social orienting by using controlled social orientating; this has important implications for prevention of and intervention in autism.

However, contrary to our original predictions, the results show that autistic traits affected reflexive social orienting only in the long SOA (400 ms) condition, not in the short SOA (100 ms) condition, a finding that seems to contradict the results with the cue-predictability manipulation. Such a contradiction was also found in a recent study on ASD children’s orienting to social interaction, in which it was found that children with ASD were able to orient to social interaction as well as children with typical development in the predictive condition, but they showed an orienting deficiency to social interaction in the non-predictive condition. More importantly, this deficiency also occurred in the long SOA (300 ms) condition, but not in the short SOA (150 ms) condition (Morrisey et al., 2018). Therefore, it is likely that different components of social information processing are sensitive to the manipulation of SOA and the manipulation of cue predictability. The manipulation of cue predictability may involve the motivational components of the initiation of processing (Chevallier et al., 2012), whereas the manipulation of SOA may involve the cognitive components related to different processing styles (Falter et al., 2013). Specifically, the non-predictive and predictive conditions may induce spontaneous and volitional initiating of processing, respectively, whereas short and long SOAs reflect automatic and effortful controlled processing, respectively. If this distinction is correct, as indicated by the results of Morrisey et al. (2018) and our study, we can speculate that the deficiency of social orienting in individuals with autism or HA is due to an insufficiency of spontaneously effortful processing (i.e., effort-controlled processing that is spontaneously initiated) of social cues, which should be one of the focal points of social attention deficit intervention.

### Contributions, Limitations, and Prospects

The present study had some theoretical and practical contributions. Firstly, by demonstrating that autistic traits could influence social orienting for of neurotypical individuals, we provided important evidence for the relationship between autistic traits and social attention, confirming the validity of the hypothesis of a continuum of autistic traits. Secondly, our study provided strong evidence for the social domain specific perspective with the founding that subjects with HA had an advantage in non-social cue (arrow) processing while they showed deficiency in social cue (gaze) processing. Thirdly, by showing that autistic traits affected reflexive social orienting only in the long SOA condition rather in the short SOA condition, the present study implicated that the deficiency of social orienting in individuals with autism might be due to an insufficiency of spontaneously effortful processing.

However, there were several limitations of our study and further researches are necessary. First of all, the effect size in our study was relatively small. This might be because the participants were divided into different groups by a median-split of their AQ scores, which resulted in the small difference in AQ scores between the two groups. Stricter grouping method can be considered in future researches. For example, participants scored at the top 10% and the low 10% in AQ questionnaire can be grouped as high autistic group and low autistic group, respectively. Secondly, multiple variables are considered simultaneously in an experiment, but it also increases the difficulty and complexity of data analysis. Base on the findings in our study, future researches can focus on the role of the manipulation of SOAs and the manipulation of cue-predictability separately. Finally, the concept of spontaneously effortful processing is just a preliminary idea, and more researches are needed to investigate its connotation, function, and relevant mechanism.

## Conclusion

The results of the present study show that compared to individuals with low autism traits, individuals with high autism traits benefit less from non-predictive social cues than from non-social ones, providing evidence that such spatial attention impairment in individuals with high autism traits is specific to the social domain. Interestingly, the smaller benefit from non-predictive social cues in individuals with HA was shown only in the 400 ms SOA condition, not in the 100 ms condition, suggesting that their difficulties in orienting to non-predictive social cues may be caused by a deficiency in spontaneously effortful control processing. These results provide strong evidence for a continuum of autistic traits in terms of social attention and have important implications for the prevention of and intervention in social attention deficiency in autism.

## Data Availability Statement

The raw data supporting the conclusions of this article will be made available by the authors, without undue reservation, to any qualified researcher.

## Ethics Statement

The studies involving human participants were reviewed and approved by the Ethics Committee of the School of Psychology at South China Normal University. The patients/participants provided their written informed consent to participate in this study.

## Author Contributions

GL and YC designed the study and carried out the experiment. GL analyzed the data, wrote the manuscript, and supervised the entire work. JZ and LH edited the manuscript.

## Conflict of Interest

The authors declare that the research was conducted in the absence of any commercial or financial relationships that could be construed as a potential conflict of interest.
